# The Operant Plantar Thermal Assay: A Novel Device for Assessing Thermal Pain Tolerance in Mice

**DOI:** 10.1523/ENEURO.0210-19.2020

**Published:** 2020-03-06

**Authors:** Ashlie N. Reker, Sisi Chen, Katherine Etter, Taylor Burger, Makayla Caudill, Steve Davidson

**Affiliations:** Department of Anesthesiology and Pain Research Center, University of Cincinnati, College of Medicine, Cincinnati, OH 45267

**Keywords:** analgesia, inflammatory pain, neuropathic pain, novel methods, operant learning

## Abstract

Pain is a multidimensional experience of sensory-discriminative, cognitive, and affective processes; however, current basic research methods rely heavily on response to threshold stimuli, bypassing the supraspinal processing that ultimately gives rise to the pain experience. We developed the operant plantar thermal assay (OPTA), which utilizes a novel, conflict-based operant task requiring evaluation and active decision-making to obtain reward under thermally aversive conditions to quantify thermal pain tolerance. In baseline measures, male and female mice exhibited similar temperature preferences, however in the OPTA, female mice exhibited greater temperature-dependent tolerance, as defined by choice time spent in an adverse thermal condition to obtain reward. Increasing reward salience (4% vs 10% sucrose solution) led to increased thermal tolerance for males but not females. To determine whether neuropathic and inflammatory pain models alter thermal tolerance, animals with chronic constriction injury (CCI) or complete Freund’s adjuvant (CFA), respectively, were tested in the OPTA. Surprisingly, neuropathic animals exhibited increased thermal tolerance, as shown by greater time spent in the reward zone in an adverse thermal condition, compared with sham animals. There was no effect of inflammation on thermal tolerance. Administration of clonidine in the CCI model led to increased thermal tolerance in both injured and sham animals. In contrast, the non-steroidal anti-inflammatory meloxicam was anti-hyperalgesic in the CFA model, but reduced thermal pain tolerance. These data support the feasibility of using the OPTA to assess thermal pain tolerance to gain new insights into complex pain behaviors and to investigate novel aspects of analgesic efficacy.

## Significance Statement

The translation of novel pain management techniques has been hindered, in part, by reliance on pre-clinical models that do not to measure the multidimensional experience of pain. Here we present a novel device and protocol to assess pain tolerance in the mouse. We show that pain tolerance is a dynamic behavior influenced by sex, that hypersensitivity does not necessarily predict pain tolerance, and that analgesics that reduce hypersensitivity may not enhance pain tolerance. This approach increases the capability to pursue new directions in basic pain research.

## Introduction

Chronic pain, as a primary condition and sequela, is a leading cause of global morbidity and disability (Rice et al., 2016). Current treatments for pain are often ineffective, possess unwanted side-effects, and carry the potential for abuse, as exemplified by the high number of opioid-related deaths (Dart et al., 2015; Rudd et al., 2016). Despite the recognition of pain relief as a major health care and research priority (Institute of Medicine, 2011), the translation of novel, non-opioid analgesics to the clinic continues to exhibit low rates of success. Myriad reasons for the lack of new, safe, and efficacious analgesics have been hypothesized (Vierck et al., 2008; King and Porreca, 2014). Here, we address the concern that the most frequently used tests of nociception in animal models, i.e., measures of reflexive withdrawal to threshold stimuli, are incomplete proxies for the human chronic pain experience.

Pain is a multidimensional experience, comprised of sensory-discriminative, affective, motivational, and cognitive components, which are generated by the brain (Melzack, 1999; Price, 2000). Given that reflexive withdrawal can occur with a latency preceding conscious perception of the stimulus (Fendrich et al., 2004; Vierck and Yezierski, 2015) and occurs in decerebrate organisms (Woolf, 1984), reflexive measures alone cannot produce a complete picture of pain processing. Alternatively, non-reflexive methods of modeling and assessing pain behaviors have been developed including, real-time and conditioned-place preference and aversion, the grimace scale, and naturalistic/home cage behaviors, which may capture additional aspects of the multidimensional components of pain (Labuda and Fuchs, 2000; Walczak and Beaulieu, 2006; King et al., 2009; Langford et al., 2010; Urban et al., 2011; Jirkof, 2014; Kandasamy et al., 2016). However, these tests do not capture the dynamic ability to endure pain to achieve a deliberate goal, or pain tolerance, a critical and familiar feature on the spectrum of human pain experience. The neural substrates of pain tolerance therefore remain poorly understood despite the potential for enhanced pain tolerance to be a clinically effective therapeutic strategy, especially for individuals coping with chronic pain.

We designed and constructed a novel, inexpensive device and developed a behavioral protocol to quantify pain tolerance in mouse models through an investigator-independent and un-biased operant task. The operant plantar thermal assay (OPTA) utilizes operant learning and decision-making within an approach-avoidance conflict paradigm to establish the duration and intensity of a noxious stimulus an animal will withstand to obtain a reward. Here we establish the parameters at which the OPTA can be used to determine baseline thermal pain tolerance. We test whether thermal pain tolerance is a dynamic behavior influenced by sex, motivation, and analgesics in neuropathic and inflammatory models of pain. We further demonstrate the effects of common analgesics on thermal pain tolerance behavior under this conflict paradigm. These experiments illustrate the utility of the OPTA as a practical tool to establish and modulate pain tolerance behavior, which can be used to complement standard threshold-level nociceptive testing.

## Materials and Methods

### OPTA: apparatus

**Figure 1.**
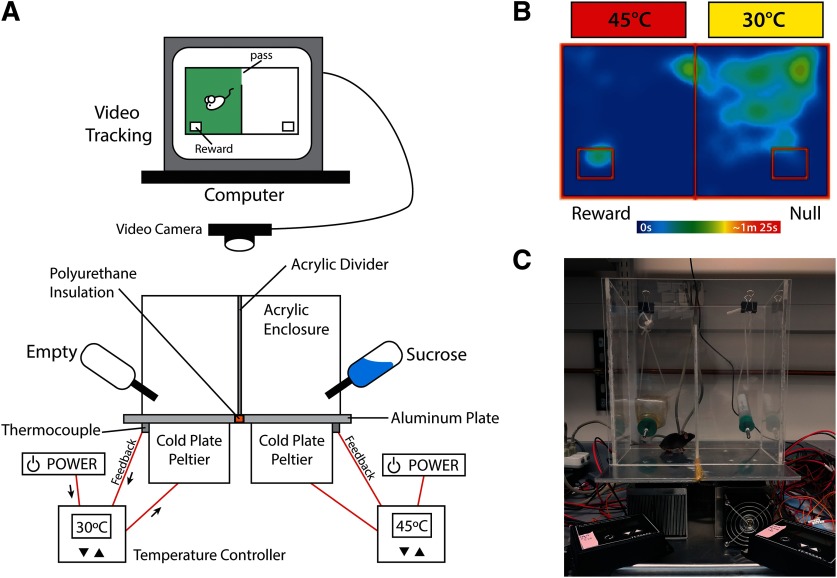
OPTA apparatus and animal tracking. ***A***, Schematic of the OPTA. Each power supply runs both a Peltier and a temperature controller. The temperature controller receives feedback from a thermode attached to the aluminum plate, allowing independent real-time thermal control. The acrylic enclosure creates a choice paradigm by isolating each floor into chambers between which a narrow pass exists. The video system tracks the head of the animal. ***B***, A representative heatmap illustrating the approach-avoidance conflict. The majority of activity is seen on the null (30°C) side, with increased activity apparent at pass point between chambers, while activity in the aversive reward side is largely limited to the reward zone. ***C***, Photograph of the OPTA in operation.

The floor of the OPTA is made of two 12” × 6” × ¼” aluminum plates (3003, MetalsDepot), which are fixed to a cold plate peltier (Cold plate cooler, CP-061, TE Technology) using MX4 Thermal Conductive Paste (Arctic) and countersunk screws (M4 machine screws), then joined using non-conductive PL Premium Polyurethane construction adhesive (Loctite). Each Peltier is independently controlled by a power supply (PS-12–8, 4A, TE Technology) and temperature controller (TC-48–20, TE Technology). A thermistor delivers real-time feedback to the temperature controller, allowing precise thermal regulation of the floor. A ¼” thick acrylic (SourceOne) enclosure surmounts the apparatus. This enclosure is divided into two equally sized chambers (5.5” × 5.5” × 12”) by placing a wall (¼” × 4” × 12”) at the midpoint of the enclosure creating a narrow, 1.5” wide passageway to allow movement between the chambers. Two equidistant holes are drilled through the rear wall of the acrylic enclosure through which the spouts of water bottles are mounted. To circumvent the potential confound of object novelty, spouts matched those used in the home cage. One bottle was empty (null zone), the other contained a 4% sucrose solution (reward zone), which is innately rewarding to mice and widely used in appetitive operant conditioning paradigms (Lewis et al., 2005). Zone areas of ∼1.75” × 2.5”, were defined within ANY-maze software (Stoelting) and positioned at the furthest distance from the entry point between the chambers. The zone area was such that only the head of the mouse could occupy the space. Video recording was from an overhead mounted camera (Logitech) using the “head-tracking” function in ANY-maze. Animals were placed in the null side at the start of each training and testing session. Location of null and reward sides remained constant through all experiments. This paradigm presents an approach-avoidance conflict wherein the animal must choose between obtaining a reward by traversing an experimentally determined aversive temperature, or forgo reward. Accumulated time in the reward zone was considered a measure of reward seeking behavior. See Figure 1 for additional apparatus details.

### Animals

Adult (8- to 12-week-old) male and female C57BL/6J mice were housed four per cage. Food and water were available *ad libitum*, except as described. Facilities were maintained at ∼22% humidity and 22°C on a 12/12 h light/dark cycle. Testing occurred during the light cycle. Animal testing procedures and handling complied with the ethical guidelines and standards established by the Institutional Animal Care and Use Committee in compliance with the Guide for Care and Use of Laboratory Animals [National Research Council (U.S.), 2011].

### Pain models

Neuropathic pain was induced using a modified chronic constriction injury (CCI) of the left sciatic nerve performed under isoflurane anesthesia, wherein three ligations were applied to the nerve using 6–0 chromic gut, until a brief twitch of the surrounding muscle was seen (Bennett and Xie, 1988; Taves et al., 2016). Control sham surgery for CCI required the nerve be only located and freed. Muscle and skin of both groups were closed with sutures or Vetbond. To establish hypersensitivity, threshold testing was performed on day 7 postoperative, coinciding with OPTA test day. Inflammatory pain was modeled by administering 10 μl of complete Freund’s adjuvant (CFA; Thermo Fisher Scientific) into the plantar surface of the left hind paw 24–48 h prior to testing, as described previously (O'Brien et al., 2015). The control for CFA was a saline injection of equivalent volume. All experiments assessing pain models used only male mice in light of initial results with the OPTA.

### Analgesic models

For all experiments, an equivalent volume of 0.9% biological saline (Hospira Inc) was injected as vehicle control. The α2-agonist, clonidine (Sigma-Aldrich) was administered intrathecally at a concentration of 0.1 μg/5 μl in a total volume of 5 μl to animals having received CCI or sham, as described previously (Hylden and Wilcox, 1980; Fairbanks, 2003). This dose did not induce motor dysfunction in mice (Stone et al., 2014). Investigators were blind to treatment for experiments measuring the effect of vehicle and clonidine in sham versus CCI. The nonsteroidal anti-inflammatory, meloxicam (Henry-Schein), was administered subcutaneously, into the nape of the neck, at 2 mg/kg (Kolstad et al., 2012). Testing was conducted in the OPTA using a 40°C reward zone. The effects of meloxicam versus vehicle on pain tolerance in animals having received CFA or control plantar injection was assessed.

### Radiant heat withdrawal assay

A modified Hargreaves test (Hargreaves et al., 1988) was used to assess threshold response to a localized radiant heat source applied to the ventral surface of the left hind paw. Animals were habituated in an acrylic box (4” × 4” × 6”) situated on top of a tempered glass surface (30” × 12” × ¼”; IITC). Animals were acclimated to testing conditions for ∼60 min, after which, a beam of radiant heat was concentrated on 4 × 6 mm spot of the plantar surface of the hind paw. Time to withdraw from the stimulus was measured as an indication of thermal threshold (Cheah et al., 2017).

### Analysis

The principle measure of interest from the OPTA was time spent in the reward zone. Distance traveled was also recorded to assess mobility; *t* tests (two-tailed), were conducted to assess differences in thermal preference, thermal threshold, reward seeking, learning and extinction, distance traveled, pooled comparisons, and time in reward zone. ANOVA was used to explore the effect of training day, impact of sex, sucrose concentration, temperature, pain, and pain with analgesia on time spent in the reward zone, thermal threshold, and distance traveled. Pearson correlation assessed time in reward zone with sucrose consumption. Where appropriate, Tukey’s or Sidak’s *post hoc* tests were conducted to determine significance. All analyses were done using GraphPad Prism 7; α = 0.05 was set as the determinant of significance. Data are reported as mean ± SEM.

## Results

### Baseline thermal preference

Side bias was assessed for female and male mice during a 10-min free exploration of the OPTA with two empty bottles present. Neither females nor males displayed a side preference (*t*_(30)_ = 0.6, *p *=* *0.55, males and females combined, data not shown). To establish the thermal preference of wild-type, naive female and male mice in the OPTA, five free choice tests were performed, wherein animals were given 900 s to freely explore the OPTA with floor temperature pairings of 15/20°C, 20/25°C, 25/30°C, and 30/35°C. A reduced test time of 600 s was set for 35/40C° to prevent the possibility of undue discomfort to the animals. No bottles were present during these tests. Preference or aversion was measured as proportion of total test time spent in each chamber. The chamber in which significantly less time was spent was interpreted as thermally aversive.

**Figure 2.**
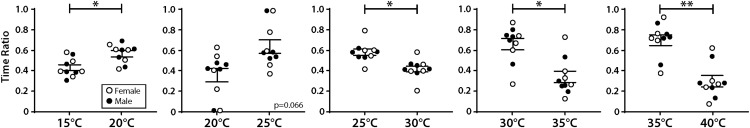
Thermal preference of adult naive wild-type mice, no reward. Naive male (*n* = 5) and female (*n* = 5) mice were monitored for 900 s (600 s for 35/40°C) on pseudo-randomly presented paired-temperature preference tests. Ratio of time spent in each chamber per test is shown. No between sex differences were detected. Paired *t* test (two-tailed). Data are presented as mean ± SEM, *p ≤ 0.05, **p ≤ 0.01.

Within sex preferences (data not shown) indicated that female mice did not prefer any temperature significantly across the five experimental conditions. Male mice displayed a preference for 25°C in the 25/30°C condition (*t*_(4)_ = 4.32, *p *=* *0.01); a preference for 30°C in the 30/35°C condition (*t*_(4)_ = 3.25, *p *=* *0.03); and a preference for 35°C in the 35/40°C condition (*t*_(4)_ = 3.05, *p *=* *0.04). Overall, male and female mice displayed the same pattern of thermal preference, with no between-sex difference. As there was no difference between sexes at any given temperature, data were combined to assess overall thermal preference (Fig. 2). Overall, 20°C was preferred to 15°C (*t*_(9)_ = 2.36, *p *=* *0.04); 25°C to 30°C (*t*_(9)_ = 2.74, *p *=* *0.02); 30°C to 35°C (*t*_(9)_ = 2.88, *p *<* *0.02); and 35°C to 40°C (*t*_(9)_ = 3.82, *p *=* *0.004). Based on these results, future experiments set the null side of the OPTA to 30°C, as the approximate midpoint between 43°C and 15°C, the thermal thresholds for acute activation of nociceptors (Bautista et al., 2007; Julius, 2013; Zheng, 2013; Tékus et al., 2016), allowing the broadest dynamic range for testing.

### Training

**Figure 3.**
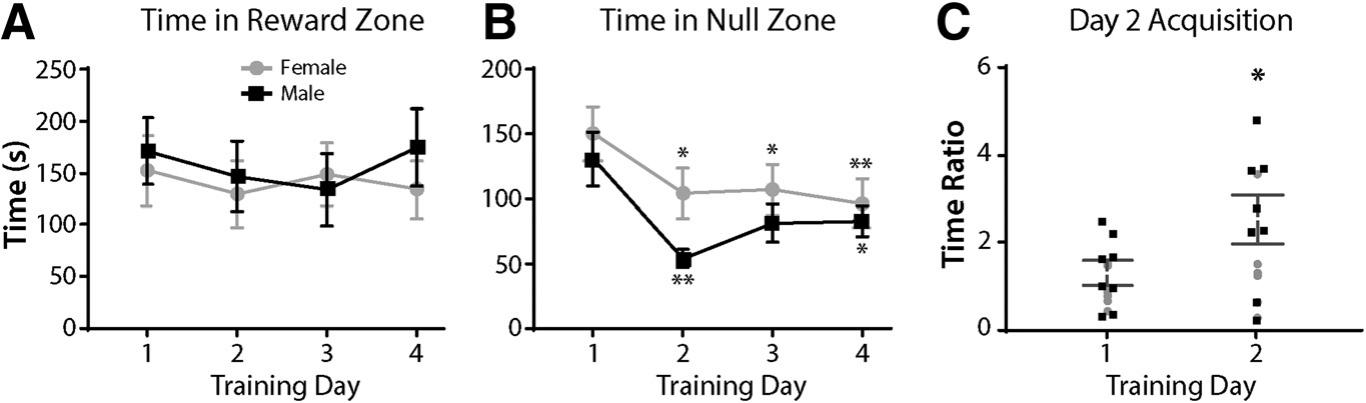
Training for operant acquisition. ***A***, Female (*n *=* *8) and male (*n *=* *8) mice showed no difference in time spent in reward zone across 4 d of training, one-way ANOVA with Tukey’s correction. ***B***, Female and male mice decreased time spent in the null zone after the first training day, one-way ANOVA with Tukey’s correction. All significances are in comparison with day 1. ***C***, Female and male mice show an increase in ratio of time spent in the reward compared with null zone on day 2 of training, unpaired *t* test (two-tailed). Data are presented as the mean ± SEM; *p ≤ 0.05, **p ≤ 0.01.

We next established the number of trials required for female and male naive mice to attain a stable baseline level of time in the reward zone (Fig. 3). Mice were food and water restricted for 4–5 h prior to a 30-min free-choice task with both floors set to 30°C and 4% sucrose available in the reward zone, while an empty water bottle was in the null zone. There were a total of four sessions, each separated by 24 h. Female mice showed no significant difference in time spent in the reward zone across 4 d of training (*F*_(2.641,18.49)_ = 0.88, *p *=* *0.457), nor did male mice (*F*_(1.714,12)_ = 1.754, *p *=* *0.215; Fig. 3*A*). However, females and males exhibited an effect of training day on time spent in the null zone (*F*_(2.198,15.39)_ = 7.128, *p *=* *0.006; *F*_(1.602,11.21)_ = 9.103, *p *<* *0.005, respectively; Fig. 3*B*). The ratio of time spent in the reward to the null zone (Fig. 3*C*) increased 60% from day 1 (M =* *1.170) to day 2 (M =* *1.965; *t*_(15)_ = 2.616, *p *=* *0.02) demonstrating a clear preference for location of sucrose reward. This indicates that mice successfully acquired and executed the operant paradigm with 2 d of training, thus future experiments included two training days.

### Temperature-dependent tolerance: sex differences and reward salience

**Figure 4.**
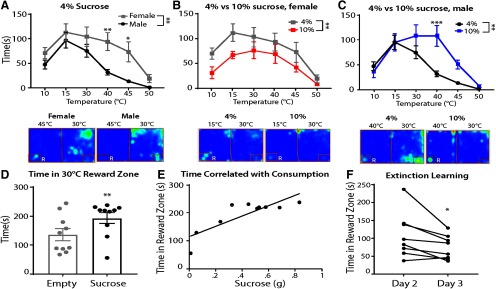
Temperature-dependent tolerance is affected by sex and reward salience. ***A***, Female (*n *=* *8) and male (*n *=* *8) mice differed in their tolerance to temperatures in the OPTA with 4% sucrose reward. Below, Representative heat maps of female and male mice in 30/45°C test. ***B***, Male mice increased tolerance to aversive temperatures when 10% sucrose was available (*n *=* *7) compared with a reward of 4% sucrose (*n *=* *8). Below, Representative heat maps of 4% and 10% sucrose conditions. ***C***, Female mice (*n *=* *8) exhibited reduced time in the reward zone when the reward was 10% sucrose compared with a reward of 4% sucrose across temperatures. Below, Representative heat maps of 4% and 10% sucrose conditions. ***D***, Male mice (*n *=* *10 per group) spend more time in reward zone when 4% sucrose is present compared with when an empty bottle is present, unpaired *t* test (two-tailed). Furthermore, time spent in reward zone is significantly positively correlated with sucrose solution consumption. ***E***, Pearson correlation, y = 185.4 + 114.8. ***F***, Upon removal of the reward, male mice spend less time in the reward zone, paired *t* test (two-tailed). Note, male and female mice in 4% sucrose condition served as sex and age matched control for 10% sucrose condition. R = reward zone. ***A–C***, Two-way ANOVA with Sidak’s correction. Data are presented as the mean ± SEM; *p ≤ 0.05, **p ≤ 0.01, ***p ≤ 0.001.

Next, we investigated thermal tolerance in naive female and male mice by testing in the OPTA for 20 min with the null side set to 30°C, and the reward side set to 10°C, 15°C, 30°C, 40°C, 45°C, or 50°C, with 4% sucrose available in the reward zone. Temperatures were presented 24 h apart, in a pseudo-random order, over 6 d. The values for each temperature were matched and means compared within and between sexes (Fig. 4*A*). There was a significant main effect of temperature (*F*_(5,70)_ = 14.62, *p *<**0.001), with all mice displaying an aversion to the extreme temperatures as indicated by less time in reward zone. A significant main effect of sex (*F*_(1,14)_ = 12.47, *p *=* *0.003) indicated that, compared with males, female mice overall spent more time in the reward zone, displaying a significantly higher tolerance to 40°C and 45°C compared with males in the OPTA (Tukey *post hoc*: *p* = 0.009 and *p *=* *0.01, respectively). Within group, females showed a specific aversion only to 50°C compared with all other temperatures (Tukey *post hoc*: *p *≤* *0.05). Males tolerated 15°C significantly more than 40°C, 45°C, and 50°C (Tukey *post hoc*: *p *≤* *0.005) and also tolerated 30°C significantly more than 45°C and 50°C (Tukey *post hoc*: *p *≤* *0.01). Representative heat maps are shown.

To assess the magnitude of reward on thermal tolerance, female and male mice were exposed to the protocol as above, however a 10% sucrose solution was used. Total time in reward zone was compared with that of the sex and age matched animals from the above experiment. Female mice exhibited less time in the reward zone under 10% sucrose conditions (*F*_(1,14)_ = 6.63, *p* = 0.02; Fig. 4*B*). Representative heat maps of 4% and 10% sucrose conditions for females are shown. In contrast, in male mice (Fig. 4*C*) the interaction effect between sucrose concentration and temperature was significant (*F*_(5,65)_ = 3.2, *p *=* *0.012), indicating that increased reward led to increased thermal tolerance. Male mice specifically spent more time in the 10% reward zone compared with the 4% zone at 40°C (*p *=* *0.002, Sidak’s *post hoc*). Representative heat maps of 4% and 10% sucrose conditions for males at 30/40°C are shown below.

We next established whether 4% sucrose was sufficiently motivating for male mice to spend time in the reward zone. Male mice spent more time in the reward zone when 4% sucrose was present relative to an empty bottle (*t*_(18)_ = 2.06, *p* = 0.05; Fig. 4*D*). A significant positive correlation between time in reward zone and sucrose consumption was observed (r_18_ = 0.83, *p *=* *0.003; Fig. 4*E*). An acquisition and extinction protocol was conducted with both floors set to 30°C. On day 1, mice were given a 10-min free exploration of the OPTA with both bottles empty to introduce the environment. This was followed 24 h later (day 2) by a 30-min acquisition period, in which 4% sucrose was available in the reward zone. Mice were returned to the apparatus 24 h later (day 3) for extinction learning wherein both bottles were empty. On day 3, removal of the reward significantly decreased reward zone time from that of day 2 (*t*_(7)_ = 2.67, *p* = 0.03; Fig. 4*F*). These data indicate that male mice were incentivized to access the reward zone to consume sucrose.

### Thermal tolerance in a model of neuropathic injury

**Figure 5.**
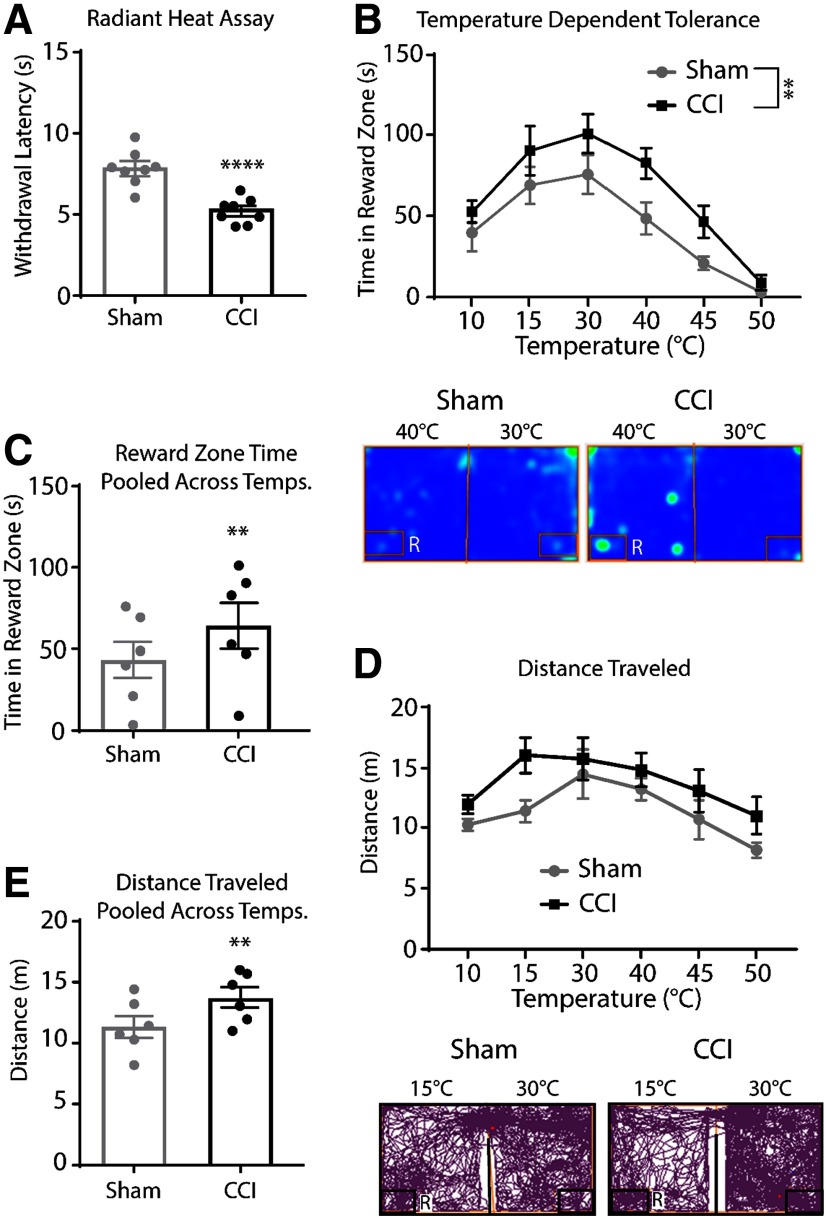
Neuropathic pain increased time in reward zone and mobility in the OPTA. ***A***, The radiant heat withdrawal assay showed decreased paw withdrawal latency in CCI versus sham (*n *=* *8 per group), unpaired *t* test (two-tailed). ***B***, High and low temperatures were aversive for both CCI and sham animals, although CCI animals spent more time in the reward zone across even aversive temperatures, two-way ANOVA, Sidak’s correction. Below, Representative heat maps at 30/40°C test. ***C***, Analysis of pooled time in the reward zone, paired *t* test, (two-tailed). ***D***, CCI presented with greater distance traveled throughout testing, two-way ANOVA, Sidak’s correction. Below, Representative track plots at 30/15°C test. ***E***, Analysis of pooled distance traveled, paired *t* test, (two-tailed). Data are presented as the mean ± SEM; **p ≤ 0.01, ****p ≤ 0.001. R = reward zone.

The radiant heat withdrawal assay was performed 7 d after CCI or sham surgery to assess the effect of neuropathic injury on thermal nociception. Mice with CCI displayed thermal hyperalgesia as indicated by reduced withdrawal latency compared with sham animals (*t*_(14)_ = 5.40, *p *<* *0.0001; Fig. 5*A*). The OPTA was used to further assess the impact of neuropathic injury on thermal tolerance (heat maps at 30/40°C below; Fig. 5*B*). We observed a main effect of temperature (*F*_(5,70)_ = 22.19, *p *=* *0.0001), with CCI and Sham groups displaying an aversion to cold and hot temperatures. There was also a main effect of CCI (*F*_(1,14)_ = 9.41, *p *=* *0.01), surprisingly, CCI animals displayed an increase in time spent in the reward zone across all temperatures. Total time spent in the reward zone across all temperatures showed that animals with CCI spent more time overall in the reward zone versus sham (*t*_(5)_ = 3.24, *p *=* *0.02; Fig. 5*C*). Total distance traveled was assessed to test for mobility differences between groups during testing (tracking plots at 30/15°C below; Fig. 5*D*). There was a trend for main effect of CCI (*F*_(1,14)_ = 4.22, *p *=* *0.06) and a main effect of temperature (*F*_(5,70)_ = 5.84, *p *<* *0.001). Total distance traveled across all temperatures showed that CCI animals exhibited increased distance traveled compared with sham (*t*_(5)_ = 4.78, *p *=* *0.01; Fig. 5*E*). Taken together, these results show that despite the presence of neuropathic injury-induced heat hyperalgesia, the capacity to tolerate even aversive temperatures was enhanced during neuropathic pain in the presence of a reward.

### Effects of clonidine in CCI model of neuropathic pain

The α2-adrenergic agonist clonidine or equivalent volume of vehicle (0.9% saline) was tested to evaluate the effect of analgesia on time in reward zone in the CCI-induced model of neuropathic pain versus sham surgery. To mitigate a potential ceiling effect and enhance detection, 45°C was chosen as the challenge temperature for testing with clonidine.

**Figure 6.**
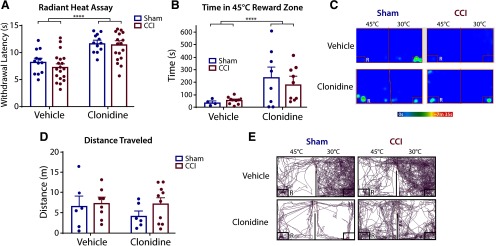
Neuropathic pain and clonidine in the OPTA. ***A***, The radiant heat withdrawal assay showed clonidine increased withdraw threshold regardless of neuropathic pain, (*n *=* *12 per group), two-way ANOVA, Sidak’s correction. ***B***, Clonidine increased time in reward zone in noxious heat chamber. Sham vehicle (*n = *4); sham clonidine (*n = *8); CCI vehicle (*n = *10); CCI clonidine (*n = *9), two-way ANOVA, Sidak’s correction. ***C***, Representative heat maps of vehicle and clonidine treatment in sham and CCI. ***D***, Neither clonidine nor CCI influence distance traveled, two-way ANOVA, Sidak’s correction. ***E***, Representative tracking plots of vehicle and clonidine treatment in sham and CCI surgeries. Sham vehicle (*n = *6); sham clonidine (*n = *6); CCI vehicle (*n = *8); CCI clonidine (*n = *10). R = reward zone. Data are presented as the mean ± SEM; ****p ≤ 0.001.

Threshold for withdrawal to radiant heat (Fig. 6*A*) was tested 7 d after CCI. Tests conducted 30–60 min after intrathecal injection showed no main effect of CCI. (*F*_(1,29)_ = 0.68, *p *=* *0.41). A main effect of clonidine (*F*_(1,29)_ = 40.25, *p* < 0.0001) was found. Both sham and CCI animals administered clonidine exhibited an increased withdrawal latency compared with vehicle (*t*_(30)_ = 6.72, *p *<* *0.001, data not shown).

When assessed for effects on thermal tolerance (Fig. 6*B*), clonidine resulted in an increase in time in the noxious reward zone in both CCI and sham animals compared with vehicle (*F*_(1,27)_ = 9.51, *p *=* *0.005), with no effect of CCI on time in the reward zone (*F*_(1,27)_ = 0.10, *p * = * *0.76). Representative heat maps are presented in Figure 6*C*. Analysis of distance traveled (Fig. 6*D*) revealed no main effect of clonidine (*F*_(1,26)_ = 0.74, *p *=* *0.40) or CCI (*F*_(1,26)_ = 1.40, *p *=* *0.25) compared with respective controls. Representative tracking plots are presented in Figure 6*E*. Taken together, these data indicate that clonidine has a robust effect on both thermal nociceptive threshold and tolerance to aversive temperature regardless of neuropathic injury state.

### Thermal tolerance in a model of inflammation

**Figure 7.**
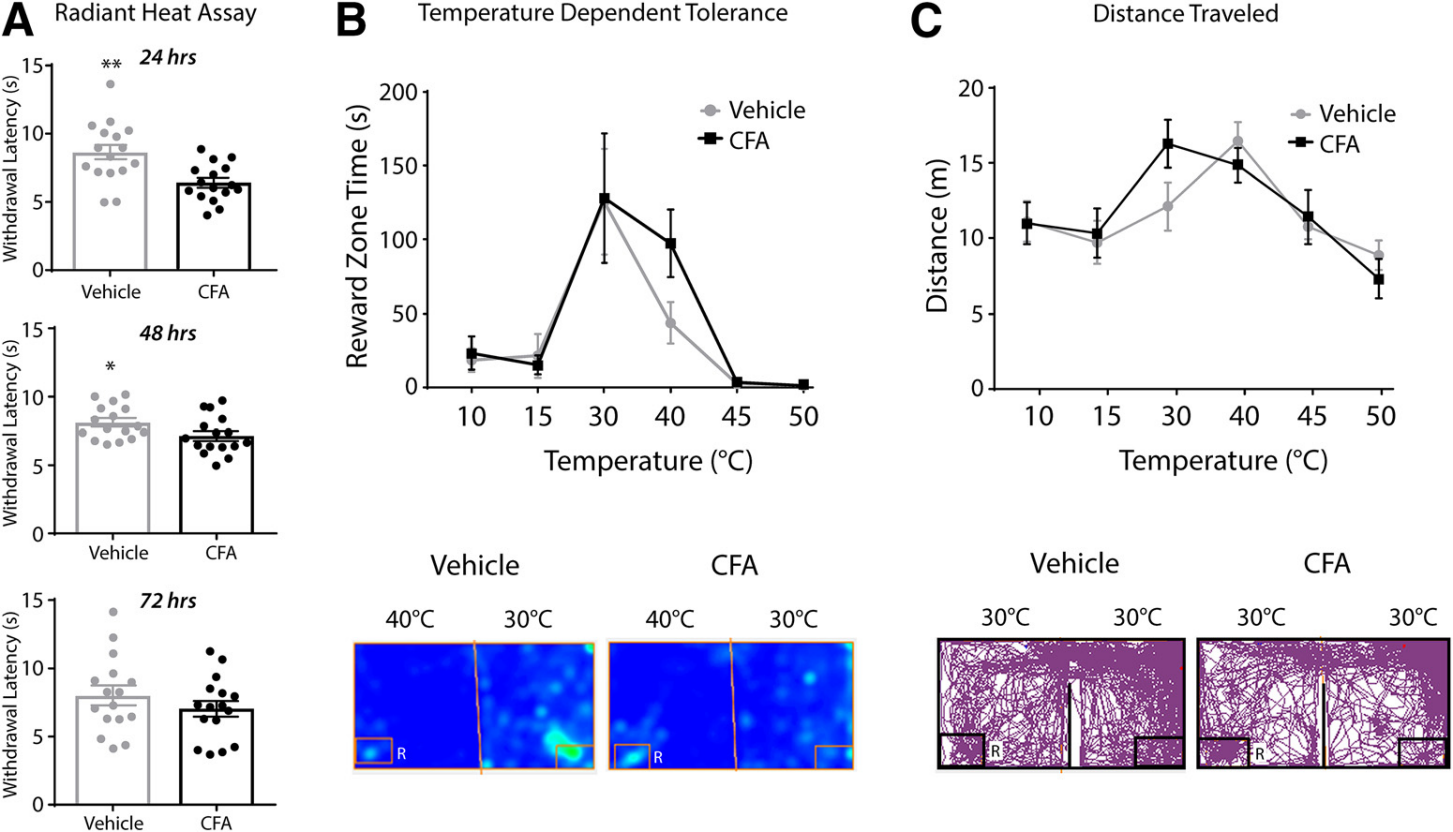
Inflammatory pain in the OPTA. ***A***, CFA (*n *=* *16) resulted in reduced withdrawal latency compared with sham (*n *=* *16) in the radiant heat withdrawal assay indicating the presence of hyperalgesia from CFA injection up to 48 h after injection, unpaired *t* tests (two-tailed). ***B***, CFA did not affect time in reward zone in the temperature-dependent tolerance test, two-way ANOVA, Sidak’s correction. Below, Representative heat maps. ***C***, CFA did not alter distance traveled at any specific temperature or across pooled temperatures, two-way ANOVA, Sidak’s correction. Below, Representative track plots. R = reward zone. Data are presented as the mean ± SEM; *p ≤ 0.05, **p ≤ 0.01.

The radiant heat withdrawal assay was conducted 24, 48, and 72 h after CFA or vehicle injection (Fig. 7*A*). The CFA group displayed significantly reduced latency to withdraw 24 and 48 h after injection compared with the vehicle group (*t*_(15)_ = 3.46, *p *=* *0.004; *t*_(15)_ = 2.62, *p *=* *0.02, respectively), but did not show reduced withdrawal latency 72 h after injection (*t*_(15)_ = 1.6, *p *=* *0.13). Subsequent OPTA testing was therefore limited to 2 d post-CFA administration.

In temperature-dependent tolerance tests, there was a main effect of temperature on time spent in reward zone (*F*_(5,52)_ = 14.53, *p *<* *0.0001), but no main effect of CFA, indicating that CFA did not modify thermal pain tolerance (*F*_(1,52)_ = 0.80, *p *=* *0.37; heat maps at 30/40°C below; Fig. 7*B*). Temperature resulted in a main effect on distance traveled (*F*_(5,52)_ = 7.53, *p *<* *0.001), but no main effect of CFA on distance was found (*F*_(1,52)_ = 0.20, *p *=* *0.65; tracking plots at 30/30°C below; Fig. 7*D*). These results indicate that within the OPTA, inflammatory state did not significantly alter the effects of temperature on reward zone time or distance traveled.

### Effects of meloxicam in CFA model of inflammation

**Figure 8.**
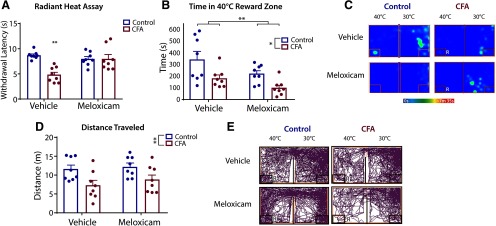
Meloxicam in a model of inflammatory pain in the OPTA. ***A***, There was a significant interaction between the effect of plantar injection and treatment on withdrawal latency in the radiant heat withdrawal assay, two-way ANOVA with Tukey’s correction. ***B***, Both CFA and meloxicam reduced time in reward zone. ***C***, Representative heat maps of vehicle and meloxicam treatment in control and CFA. ***D***, CFA resulted in reduced distance traveled as compared with control, two-way ANOVA with Tukey’s correction. ***E***, Representative track plots of vehicle and meloxicam in control and CFA. For all groups, *n* = 8. R = reward zone. Data are presented as the mean ± SEM; *p ≤ 0.05, **p ≤ 0.01.

The effects of meloxicam, or equivalent volume of vehicle (0.9% saline), were tested to evaluate the effect of analgesia on thermal threshold and time in reward zone at 40°C in the CFA-induced model of inflammatory pain. This temperature was chosen as it resulted in the greatest difference in temperature dependent tolerance tests in CFA versus control animals (Fig. 7*B*). Threshold for withdrawal to radiant heat (Fig. 8*A*) 40–80 min after treatment showed an interaction effect between plantar injection and treatment (*F*_(1,28)_ = 12.03, *p *=* *0.002), indicating that the effect of treatment on withdrawal depended on the type of injury. Specifically, CFA animals with vehicle treatment displayed decreased withdrawal time compared with control animals with vehicle treatment (*p *=* *0.0002, Tukey’s *post hoc*), control animals with meloxicam treatment (*p *=* *0.003, Tukey’s *post hoc*), and CFA animals with meloxicam treatment (*p *=* *0.002, Tukey’s *post hoc*). This indicates that the anti-hyperalgesic properties of meloxicam are apparent in the presence of inflammation only, and no additional reduction in hypersensitivity was seen as a result of meloxicam treatment in control animals as compared with vehicle treatment in control animals.

When assessed for thermal tolerance using the OPTA (Fig. 8*B*), there was a main effect for plantar injection (*F*_(1,28)_ = 6.507, *p *=* *0.02) as well as treatment (*F*_(1,28)_ = 12.25, *p *=* *0.002). Representative heat maps are presented in Figure 8*C*. Overall, CFA-treated animals spent less time in the reward zone (M =* *32.86, SEM =* *9.58) than control animals (M =* *65.74, SEM =* *14.39), and meloxicam-treated animals spent less time in the reward zone (M =* *37.31, SEM =* *14.04) than vehicle-treated animals (M =* *61.28, SEM =* *18.84). Specifically, control animals with vehicle treatment spent more time in the reward zone than both CFA animals given vehicle (*p *=* *0.04, Tukey’s *post hoc*) and CFA animals given meloxicam (*p *=* *0.001, Tukey’s *post hoc*). Analysis of distance traveled (Fig. 8*D*) revealed a main effect of injection (*F*_(1,28)_ = 11.05, *p *=* *0.003), with CFA-injected mice traveling less (M =* *10.05, SEM =* *0.99) than controls (M =* *14.89, SEM =* *0.41). Specifically, vehicle-treated CFA animals traveled significantly less than meloxicam-treated control animals (*p *=* *0.03, Tukey’s *post hoc*). Representative track plots are presented in Figure 8*E*. This indicates untreated CFA reduced mobility, but meloxicam itself does not affect mobility, thus likely does not account for reduced time in reward zone by meloxicam-treated animals. Overall, these data suggest that in the CFA model of inflammation, meloxicam is efficacious in reversing localized thermal hyperalgesia resulting from CFA, but is not effective in reversing or attenuating reduced thermal tolerance and may even promote lowered pain tolerance.

## Discussion

Unsatisfactory translation of reflexive measures of nociception, in which the endpoint is withdrawal to a threshold stimulus, has led to a crisis and demand for new models to assess non-reflexive measures of pain in animals (Mogil, 2009; Mogil et al., 2010; King and Porreca, 2014; Vierck and Yezierski, 2015; Klinck et al., 2017). Pain tolerance is an integral component of the pain experience which has been defined as “the maximum intensity of a pain-producing stimulus that a subject is willing to accept in a given situation” (Loeser and Treede, 2008). Pain tolerance is a critical limiting factor in the ability of chronic pain patients to complete daily tasks, and selectively modulating this factor is a potential alternative approach to pain management. The underlying cells and circuits mediating pain tolerance have not been identified, but evidence suggests they overlap with supraspinal processes that subserve attention, response selection, and mood (Tölle et al., 1999; Tang et al., 2008).

Operant tests targeting supraspinally regulated affective, cognitive, and motivational processing of pain are increasingly used in pain research, including conditioned preference, place escape/avoidance, the mechanical conflict system (Harte et al., 2016), and the operant orofacial pain assay (Neubert et al., 2005). While these assays provide new and important ways to investigate animal behavior, the OPTA generates information not captured by these tests. Only the operant orofacial assay, like the OPTA, produces a measure of choice time engaged with an aversive stimulus. This more closely represents the daily choices an individual with chronic pain must make wherein repeated or lengthy engagement with known aversive actions must occur to achieve life goals. However, the operant orofacial assay demands greater training and nutrient deprivation, requires that animals be nude or shaved and therefore lack whiskers, a primary source of sensation, and is focused on the trigeminal system and therefore may not be appropriate for the more common spinally mediated models of pain.

The OPTA reward floor, set at temperatures above 35°C or below 25°C, generated avoidance behavior indicative of an unpleasant somatosensory, affective, and motivational experience. Cutaneous thermosensation at somatic contact points with the floor is mediated by a heterogeneous population of primary afferent Aδ and C fibers, including thermosensitive nociceptors which activate at ≤ 28°C for cold and ≥ 37°C for heat (Green and Akirav, 2010; Schepers and Ringkamp, 2010). The central projections of these primary afferent fibers converge onto spinal neurons, including those forming the spinothalamic tract, which respond to thermal stimuli as well as noxious chemical and mechanical stimuli (Burstein et al., 1991; Zhang et al., 2006). Therefore, the aversive floor temperatures used in this study are likely encoded via thermo-nociceptive peripheral and central pathways that reach the brain.

Both male and female mice exhibited similar baseline preferences for temperature when no reward was present. Male and female mice also similarly acquired the operant paradigm during training in the OPTA. Interestingly, in the OPTA during challenge, female mice demonstrated increased heat pain tolerance compared with males. This previously unrecognized observation of sex differences on pain tolerance in mice does not match a human psychophysical study that showed added incentives to tolerate pain in the cold-pressor test increased tolerance similarly in both men and women (Lowery et al., 2003). This discrepancy could reflect differences in tolerance to heat versus cold, or species differences. In humans, females tend to exhibit lower tolerance scores than males on both cold pressor and contact heat tolerance tests, when conducted without manipulation of motivating factors (Fillingim et al., 2009; Forsythe et al., 2011; Bartley and Fillingim, 2013). However, female subjects showed enhanced heat adaptation on repeated trials compared with males (Hashmi and Davis, 2009, 2010), suggesting that females may exhibit greater tolerance to repeated heat, consistent with female mouse data from the OPTA.

Overall, female mice tend to exhibit higher sensitivity and more severe and lengthened responses to pain, and this is generally consistent with humans (Hurley and Adams, 2008). Inflammation affects afferent fibers in females more than males, specifically C-fibers which result in increased nociceptive sensitivity (Rosen et al., 2017; Sorge and Strath, 2018). Neuropathic and inflammatory models have indicated that sex differences exist in neuroimmune and hormonal interactions that can lead to variable differences in pain behaviors (Sorge et al., 2015; Taves et al., 2016; Rosen et al., 2017). Of note, pre-clinical research regarding sex differences in pain tolerance is currently lacking. Our observations indicate that pain tolerance and hypersensitivity are regulated by separate mechanisms, and offer a path to investigate the neural processing of novel pain-related behaviors. Further investigation of pain tolerance to produce a better understanding of the dependence of pain coping capacity on sex, stimulus modality, and motivational cues is needed.

To determine whether ongoing pain modifies pain tolerance behavior, the OPTA was used to test mice with CCI-induced neuropathy. This unexpectedly revealed that neuropathic mice showed greater reward time and tolerance to aversive temperatures than sham. Previous animal studies of reward-seeking behavior under neuropathic conditions have shown mixed effects with some reporting a loss of reward-seeking behavior, or anhedonia (Goffer et al., 2013; Dellarole et al., 2014; Lee et al., 2015), or no change in motivational responses to reward (Urban et al., 2011; Okun et al., 2016). Neuropathic pain can alter descending inhibitory and facilitatory systems to alter threshold nociception (Ossipov et al., 2014; Patel et al., 2018; Chen and Heinricher, 2019), but how these systems might regulate pain tolerance is still unclear. Pain-evoked plasticity of neuromodulatory signals in reward circuits has implicated the nucleus accumbens and limbic forebrain structures in nociceptive responses (Sagheddu et al., 2015; Massaly et al., 2019). These and other supraspinal regions may contribute to the decision-making processes that occur during the OPTA. In contrast to the neuropathic model, CFA-induced inflammation did not alter time in the reward zone, or thermal tolerance, suggesting that the duration, intensity, or type of injury may be an important factor in pain tolerance regulation.

We hypothesized that analgesics may work differently on pain tolerance and nociceptive thresholds. Clonidine is often used in research models of neuropathic pain, due to its analgesic efficacy and lack of innate reward (King et al., 2009). We confirmed that clonidine reduced hypersensitivity to radiant heat, and additionally found that clonidine enhanced thermal tolerance in the OPTA. However, this enhanced thermal tolerance was present in both CCI and sham animals indicating that clonidine is a drug with effects that are not specific to pain, but rather a general suppression of somatosensation. Clonidine also possess sedative effects, but the lack of a reduced distance traveled by clonidine-treated mice indicates this was unlikely to be a factor here. Dry mouth and thirst have been reported as side effects of clonidine and cannot be ruled out as an influence of time in reward zone, although this is unlikely to result from intrathecal administration.

Consistent with the literature, CFA produced thermal hyperalgesia, that was attenuated by the NSAID meloxicam, without affecting mobility (Kolstad et al., 2012). In light of these results, it was surprising that pain tolerance was reduced in both CFA and control animals treated with meloxicam, as this suggests a decreased thermal tolerance mediated by the drug itself. Given there was no main effect of meloxicam on distance traveled, it is unlikely that the drug affected choice time in reward zone. While nausea cannot be ruled out as a contributing factor to reduced time in reward zone, meloxicam has a low gastrointestinal side effect profile (Ingrao et al., 2013) which is even less likely to occur with a single subcutaneous administration.

Taken together, these results suggest that while some NSAIDs exhibit anti-hyperalgesic properties, they may also limit the capacity to tolerate pain in some cases. Psychophysical studies in humans have shown mixed results of NSAIDs on pain tolerance. The cold pressor test failed to show that NSAIDs enhance tolerance (Jones et al., 1988), although enhanced tolerance was observed with NSAIDs in a burn model (Sycha et al., 2003). Meloxicam may act within the peripheral and central nervous system (Burian and Geisslinger, 2005; Novakova et al., 2014). In addition to the well-known anti-inflammatory effects of NSAIDS which we observed, NSAIDs also have been shown to modulate descending control of the spinal cord by preferentially disrupting C-fiber input while leaving Aδ-nociceptor input intact (Waters and Lumb, 2008; Leith et al., 2014). It is possible that selective C-fiber suppression could lead to a relatively enhanced Aδ-mediated signal which consequently could limit pain tolerance.

In addition to traditional analgesics, sucrose itself has been reported to elicit analgesic effects, which may have contributed to some of the results. However, these effects are largely found only in pediatric subjects with varying efficacy and would not be expected to interfere with the decision to cross the reward side floor which precedes sucrose consumption (Kakeda, 2010; Slater et al., 2010; Wilson-Smith, 2011; Shahlaee et al., 2013).

Using the OPTA, we have identified previously uncharacterized, non-reflexive behavioral outputs of neuropathic and inflammatory pain models in male mice. Our observations indicate that hypersensitivity does not predict decreased pain tolerance, and that analgesics that reduce hypersensitivity may not necessarily enhance pain tolerance. Affective, cognitive, and motivational processing required for pain coping is thought to be mediated through higher-order neurons in regions such as the anterior cingulate, amygdala, and nucleus accumbens (Apkarian et al., 2005; Lee and Tracey, 2013). These regions contribute to a network that dictates avoidance behaviors, catastrophizing, and pain fear, which are fundamentally distinct from threshold reactions and are critical to the chronification of pain (Nees and Becker, 2018). An important modulator of these networks may be the type or duration of injury, as animals with neuropathic injury exhibited altered pain tolerance, while animals with inflammatory injury exhibited no change in pain tolerance. The CCI neuropathic model is more somatically extensive and longer lasting than CFA inflammatory model and likely results in greater recruitment and enhanced plasticity of pain-related circuitry which, in turn could generate a more robust compensatory response from descending pain-inhibitory pathways (Ossipov et al., 2014). A major goal of this study was to develop a complimentary tool to open new investigations into the supraspinal systems that contribute to pain tolerance. The OPTA can establish baseline thermal tolerance, measured as choice time in a thermally aversive reward zone, and can be used to examine how this thermal tolerance is altered in models of injury, psychiatric disorders, stress, or disease. Currently, findings in this study regarding pain and analgesia can only be applied to male mice. The unexpected divergence between males and females in temperature dependent tolerance indicates that female mice, despite possessing similar thermal preferences to males, require different testing conditions, a pursuit of future experiments. The OPTA was designed to test mice; however, minor modifications can be made to accommodate larger rodents. It is worth noting that the OPTA is not suited for pain models presenting with severely reduced mobility.

Pain tolerance is highly plastic within individuals and is altered by stress, exercise, drug use, age, race, and social situations (Rhudy and Meagher, 2000, 2003; Edwards et al., 2001; Kállai et al., 2004; Shavers et al., 2010; Lautenbacher et al., 2017; Merkle et al., 2018; Sluka et al., 2018). Longitudinal studies may be particularly useful in identifying critical time points to better understand the development of altered pain tolerance after injury. Ultimately, the OPTA can be used in tandem with many techniques in neurobiology including optogenetics and chemogenetics to identify and manipulate the neuroanatomical substrates that regulate pain tolerance in the brain. Preclinical testing of pain tolerance may lead to novel efficacious and cost-effective strategies for pain management in patients.

## Synthesis

Reviewing Editor: Karen Davis, Krembil Research Institute and University of Toronto Decisions are customarily a result of the Reviewing Editor and the peer reviewers coming together and discussing their recommendations until a consensus is reached. When revisions are invited, a fact-based synthesis statement explaining their decision and outlining what is needed to prepare a revision will be listed below. The following reviewer(s) agreed to reveal their identity: Lakeisha Lewter.

In this paper, the authors introduce a novel assay for studying thermal pain tolerance in mice termed, the Operant Plantar Thermal Assay (OPTA). This novel approach utilizes an approach-avoidance conflict paradigm, operant learning, and active decision-making, to measure thermal pain tolerance. Specifically, the authors describe a modified thermal choice assay that incorporates a sucrose-based reward (sucrose solution) in the test chamber under the premise that the presence of the reward will encourage mice to enter thermally non-preferred chambers.

The thermal assay described by the authors is well designed and may offer insights into motivational aspects of behavioural responses to thermal stimuli under normal and pathological conditions. The study is straightforward with a clear objective and appropriate controls, uses two different models of pain (inflammatory and neuropathic) and two different analgesics (clonidine and meloxicam) to investigate thermal pain tolerance in ongoing pain. The validation of the assay across both sexes of mice and in different pain models is appropriate.

However, several aspects of the study detract from the authors’ main claim that this model allows the measure of pain ‘tolerance’ and can reveal changes in pain tolerance in pain models. This and other major concerns may limit the impact of the work.

Major comments:

1. In Figure 2, the authors show the time spent in the reward zone and the null zone over 4 consecutive trials at equal temperatures per zone. The relative increase in time spent in reward zone after day 2 compared to null is interpreted as reward learning and reward ‘seeking’, which is necessary to establish for all following experiments. However, there is no change in the time spent in the reward zone over time, only a decrease in the null zone time which could be expected because there is nothing in the null zone to explore (it’s an empty bottle) and mice can presumably learn this. Therefore, the RELATIVE time in null vs reward might serve as a measure of reward learning; but because there is no change in the total time spent in the reward zone this behavioural measure is difficult to interpret alone. Some suggestions may clarify this:

a) The value for reward zone time is total time over 30-min. Is there evidence that this time increases over the time of the first or second trials? This may serve as evidence of reward learning. Similarly, is there a decrease in the time spent in the null zone in the first trials?

b) Do the results still hold when a relative difference score or ratio of null vs reward is computed?

c) The definition of “reward” seeking and reward consumption is not well defined in this study. i) How was the size and placement of the reward zone defined in Ethovision? ii) Did the authors investigate sucrose consumption in addition to reward zone approach? This metric would provide more concrete evidence that mice are consuming the ‘reward’ rather than simply approaching and exploring a potentially novel spout or corner of the assay in the test chamber.

2. It is unclear what relevance of the measure of time spent in the reward zone has to “pain tolerance”, as there is no comparable measure of how aversive the temperature is without a reward present for each animal. The interpretation of the data as ‘tolerance’ would be clearer if there was a measure of how much MORE time mice would spend in aversive chambers in the presence of a reward. This is important as the assay relies on the untested assumption that sucrose increases the amount of time spent in non-preferred temperatures. The 4% vs 10% sucrose data do not necessarily demonstrate a ‘dose’ effect of sucrose reward value because i) these are different animal cohorts, and there are cohort effects in the temperature vs reward time behaviour, and ii) the effects are opposite between sexes.

3. Without a direct measure of how the presence of the reward influences thermal preference, I do not think the authors can support their conclusion in the discussion that “upon presentation of the reward in the OPTA, female mice demonstrated increased pain tolerance compared to males”. This statement is presumably based on a comparison of the data presented in Figure 2 and 4A; yet these are different kinds of tests and are not directly comparable. It may be beneficial to investigate additional parameters in the authors current dataset that could support their discussion of tolerance. How does the time spent in the reward zone (or reward consumption) compare to the total time spent in the reward side (maybe more exploration related) and/or the number of cross-over episodes per side, and does this change according to condition?

Overall, It is difficult for this reviewer to agree that there is a clear measure of ‘tolerance’ presented in this study, as it is plausible that the same data could been acquired in the absence of reward and simply reflect thermal preference.

4. The CFA +/- meloxicam data shown in figure 8 should have been analyzed with a two-way ANOVA to analyze the 2x2 design and data should be presented similarly to the CCI +/- clonidine data presented in Figure 6, which uses the same 2x2 design.

5. It is not clear what the reader should “take-away” from Figure 3. Figure 3A displays that female and male mice showed no difference in time spent in the reward zone across 4 days of training. However, Figure 3B, shows that female and male mice decreased time spent in the null zone by day 2. If there is a decrease in time spent in the null zone on training days 2-4 (and there are only two sides of the assay), shouldn’t there also be an increase in time in reward zone across the training days in Figure 3A? Additionally, it is not clear which groups are significantly different in Figure 3C. The figure legend for 3C states that both female and male mice spend significantly more time in the reward zone compared to the null zone on day 2. However, the S.E.M error bars of the gray bars (female/reward and female/null) are overlapping.

Minor comments:

1. The discussion of clonidine refers to central effects of clonidine such as ‘dry mouth or thirst’. These are presumably greatly reduced or absent after intrathecal administration.

2. Avoid using the term ‘coping’ or describing mouse behaviour in this assay as evidence of pain ‘coping’.

3. The use of the word “rodents” in the title and throughout the manuscript is incorrect because no other species of rodent was tested beyond mice and it is not clear whether this assay is generalizable to rats or other rodents. Replace rodents with ‘mice’.

4. The discussion should include specific examples of non-reflexive and/or affective measures of pain in animals (e.g., placed-escape avoidance paradigm - PEAP; nesting behavior) and include their “shortcomings” in assessing the different components of pain, thus highlighting a need for this novel assay.

5. It is not clear why a 45C reward zone was chosen to assess the effect of clonidine on pain tolerance in CCI-induced mice, especially since there is such a strong aversion in pain-free mice with 40C (Figure 2).

6. It is not clear how side-bias was accounted for. Please include a statement stating whether side-bias was taken into account (e.g., “the reward was randomly placed on either side of the apparatus).

7. Please justify the exclusive use of male mice in the analgesic studies. Please include statements in the discussion noting that the results from the analgesic studies may be limited to males only since females were not tested. It is suggested that known differences in male and female mice also be included in discussion with regards to neuropathic/inflammatory pain.

8. Consider including a statement stating the reason for performing some studies 5 days after CCI, and other studies 7 days after CCI.

9. Please include the vehicle used for each drug.

10. In Figure 5A, the asterisks are located above the sham group, showing the sham group to have a significantly increased withdrawal latency (s) than the CCI group. It is suggested that instead, asterisks are put on top of the CCI bar, indicating that the withdrawal latency is significantly decreased in CCI-mice compared to the sham group.

11. Some temperatures were described as being “aversive temperatures“, but this aversion was not defined. Please include a statement describing what was considered aversion (e.g. Aversive temperatures were revealed when mice spent significantly less time in the reward zone).

12. It might be worthwhile for the authors to include current limitations and/or future experiments. What current limitationsexists regarding OPTA? What current knowledge gaps regarding pain can be addressed by utilizing OPTA? As a result of the findings from this study, what future experiments should be done?

13. Please add a statement regarding experimental blinding of treatment (e.g. sham vs CCI

14. Please add a statement about how the doses of Clonidine and Meloxicam were determined for the experiments assessing analgesia (e.g., this particular dose has been shown to attenuate thermal hypersensitivity devoid of sedation).
